# Exploring Factors for Predicting Anxiety Disorders of the Elderly Living Alone in South Korea Using Interpretable Machine Learning: A Population-Based Study

**DOI:** 10.3390/ijerph18147625

**Published:** 2021-07-18

**Authors:** Haewon Byeon

**Affiliations:** Department of Medical Big Data, College of AI Convergence, Inje University, Gimhae 50834, Korea; bhwpuma@naver.com; Tel.: +82-10-7404-6969

**Keywords:** explainable artificial intelligence, machine learning, stacking ensemble, Self-Rating Anxiety Scale, multiple risk factors

## Abstract

This epidemiological study aimed to develop an X-AI that could explain groups with a high anxiety disorder risk in old age. To achieve this objective, (1) this study explored the predictors of senile anxiety using base models and meta models. (2) This study presented decision tree visualization that could help psychiatric consultants and primary physicians easily interpret the path of predicting high-risk groups based on major predictors derived from final machine learning models with the best performance. This study analyzed 1558 elderly (695 males and 863 females) who were 60 years or older and completed the Zung’s Self-Rating Anxiety Scale (SAS). We used support vector machine (SVM), random forest, LightGBM, and Adaboost for the base model, a single predictive model, while using XGBoost algorithm for the meta model. The analysis results confirmed that the predictive performance of the “SVM + Random forest + LightGBM + AdaBoost + XGBoost model (stacking ensemble: accuracy 87.4%, precision 85.1%, recall 87.4%, and F1-score 85.5%)” was the best. Also, the results of this study showed that the elderly who often (or mostly) felt subjective loneliness, had a Self Esteem Scale score of 26 or less, and had a subjective communication with their family of 4 or less (on a 10-point scale) were the group with the highest risk anxiety disorder. The results of this study imply that it is necessary to establish a community-based mental health policy that can identify elderly groups with high anxiety risks based on multiple risk factors and manage them constantly.

## 1. Introduction

Anxiety, which is defined as a disorder causing difficulties in daily life due to excess worry, fear, and hyperarousal, is known as one of the most common mental disorders worldwide [1]. It was reported that one in five Americans suffered from anxiety disorders [2] and the lifetime prevalence of anxiety disorders was 9.3% in South Korea [3]. The number of patients with an anxiety disorder is rapidly increasing in South Korea: the number of patients treated for an anxiety disorder increased from 533,619 in 2014 to 690,735 in 2018, a 29.4% increase in five years [3]. Particularly, the incident rate of anxiety disorders by age group showed that the number of treated patients per 100,000 increased the most (15% increase) from 2014 to 2018 in the elderly group (≥60 years old), and the result suggested that the elderly experienced anxiety frequently and that anxiety disorder was a rapidly increasing mental illness.

A number of epidemiologic studies [3,4,5] have reported that the prevalence of anxiety disorders in the elderly is lower than that of the young/prime-aged. In particular, Gum et al. (2009) [5] examined a community-based epidemiologic survey and showed that the prevalence of anxiety disorders was 20.7% in the 18–44 years old group, 18.7% in the 45–64 years old group, and 7.0% in the 65 years old or older group, indicating that that of the elderly was the lowest. However, it is believed that the actual prevalence of anxiety disorders in the elderly may be higher than the reported, when considering the fact that the elderly are reluctant to recall and report psychiatric symptoms or often tend to express the symptoms in physical terms [6]. The elderly are at very high risk of experiencing anxiety because (1) they face a lot of social stress such as bereavement, retirement, economic hardship, and abuse from people around them, (2) they are vulnerable to anxiety due to neuro-biological changes in the brain as a result of aging, (3) they are more likely to experience the fear of death in the senescence, and (4) they suffer from more physical diseases than younger people and are taking a lot of drugs [7]. Nevertheless, since the elderly perceive emotional problems such as depression and anxiety as a result of aging and they do not seek medical assistance actively, a small number of them are diagnosed with an anxiety disorder and treated [8]. Anxiety disorders can be treated by drugs, using anti-anxiety drugs such as buspirone, or psychotherapy [9]. Therefore, it is important to identify factors associated with anxiety and detect and manage people who are very vulnerable to anxiety as soon as possible.

It is highly likely that anxiety is affected by social factors as well as the physical and psychological problems of individuals [10]. Therefore, it is necessary to consider environmental factors such as social factors and social networks, in addition to sociodemographic characteristics, when identifying factors related to anxiety. It is unavoidable that the capability to emotionally cope with social and environmental changes is more vulnerable in old age, when people tend to be highly dependent on social factors in terms of economic, physical, and mental health [11,12]. Moreover, the risk factors of anxiety are complex and more likely to cluster with each other [11,12]. Therefore, it is important in public health science to understand the characteristics of anxiety in old age, considering that South Korea is facing a super-aged society. It is clear that the elderly are vulnerable to anxiety and anxiety disorder is a common disease in the elderly. However, only a few studies have evaluated the risk factors of anxiety disorder in old age while considering social factors and social network as well as sociodemographic characteristics and personal characteristics compared to other mental disorders, such as cognitive disorders [7].

Many recent studies [13,14] have used machine learning based on big data to identify the risk factors of a disease while considering multiple risk factors. However, employing a single machine learning technique may show lower prediction performance, depending on the used algorithm, and it is possible to induce errors because the bias existing in each algorithm can affect the prediction result. For example, a decision tree model such as Iterative Dichotomiser 3 (ID3) is very useful for making simple decisions, however, when tree models are complicated, it has lower prediction power and it poses a risk of result instability (possibility of deriving different results in iterated analysis) [15]. As an alternative method to overcome this limitation, many studies have developed predictive models using various machine learning techniques and combined them into a stacking ensemble learning model to reduce the risk of bias that individual models may have [16,17,18].

On the other hand, when developing a predictive model using medical data, explanatory power (interpretation) of the results is important in addition to accuracy. Recently, one important issue in medical artificial intelligence (AI) is to develop eXplainable Artificial Intelligence (X-AI) that can explain and present decisions made by AI in a form that can be understood by humans [19]. In the case of image classification, which is unstructured data, new methods such as learning deep explanation or gradient-class activation map (Grad-CAM) have been developed and used in various fields [20]. In the case of structured data, such as examination data, Carvalho et al. (2019) [21] and Wang et al. (2019) [22] introduced a method of presenting the key predictors derived from machine learning with decision tree visualization as an alternative way to increase the interpretability of the black box model. This epidemiological study aimed to develop an X-AI that could explain groups with a high anxiety disorder risk in old age. To achieve this objective, (1) this study explored the predictors of senile anxiety using base models and meta models. (2) This study presented decision tree visualization that could help psychiatric consultants and primary physicians easily interpret the path of predicting high-risk groups based on major predictors derived from final machine learning models with the best performance.

## 2. Materials and Methods

### 2.1. Data Source

This study is a secondary data use study using the Korean Psychosocial Anxiety (KPA) Survey, a national survey. The KPA survey was conducted from August to September 2015 under the supervision of the Korea Institute for Health and Social Affairs. This study stratified 17 cities and provinces in South Korea using the population data of the statistical yearbook (complete enumeration) of the Ministry of Safety and Public Administration as of June 2015, and sampled by using the quota sampling method while considering the composition ratios of gender, age, and residential region. This study selected 200 eup, myeon, or dong for sampling sites using the probabilities proportional to size (PPS) method by treating 3552 eup, myeon, or dong in South Korea as the population. This study applied PPS after sorting cities, counties, and districts based on the administrative district code to secure the randomness of the samples. After choosing 200 sample sites, we visited the selected sample sites and chose the fifth household from the community center of each eup, myeon, and dong. As a result, this study surveyed 7000 adults who were 19 years or older. A surveyor who received survey training visited the sample household and conducted a 1:1 survey based on a computer assisted personal interview. This study was approved by the Clinical Research Ethics Committee of University H (No. 20180042). This study analyzed 1558 elderly (695 males, and 863 females) who were 60 years or older and completed the Zung’s Self-Rating Anxiety Scale (SAS) [23], which was translated into Korean and standardized.

### 2.2. Measurement and Definition of Variables

The anxiety disorder, an outcome variable, was measured using the Korean version of SAS [23], which is a translated and standardized version of Zung’s SAS [24]. SAS is a self-reporting test that encompasses emotional and psychophysiological aspects. It is a widely used standardized screening test that can easily measure anxiety disorders in healthy people [25]. The SAS consists of a 4-point Likert scale composed of 20 items, and the total score is 80 points. A higher score indicates more severe anxiety symptoms. When developing the Korean version of SAS, the Cronbach alpha value, indicating internal consistency, was 0.96, and the overall accurate discrimination rate, discriminating between healthy patients and patients with anxiety, was 93.7% [24]. In this study, the threshold of the anxiety disorder was set as 45 points.

Referring to previous studies [26,27,28,29,30], explanatory variables of this study included age, self-esteem, alcohol use disorder (normal drinker, high-risk drinker, or alcohol use disorder), subjective loneliness (very rare, occasionally lonely, often lonely, or mostly lonely), the experience of suicidal urge over the past year (yes or no), subjective frequency of communication with neighbors and friends (10-point scale; a higher score means more frequent communication), subjective frequency of communication with other family members (10-point scale), subjective satisfaction with help (support) from neighbors (yes or no), regular club activities (yes or no), perceived social support, subjective trust satisfaction with neighbors (yes or no), subjective satisfaction in the safety level of the neighborhood (yes or no), subjective satisfaction in the living environment of the neighborhood (yes or no), subjective satisfaction in the medical service of the region (yes or no), mean monthly household income (<KRW 2 million, ≥KRW 2 million and <KRW 3 million, or ≥KRW 3 million), the highest level of education (middle school graduation or less, or high school graduation or more), residential area (urban or rural), subjective satisfaction with the public transportation environment in the neighborhood, job/income instability (10-point scale), instability of preparation for old age (10-point scale), living safety instability (10-point scale), physical health instability (10-point scale), cognitive health (e.g., dementia) instability (10-point scale), family relationship and dissolution instability (10-point scale), instability in family support and caregiving (10-point scale), instability in relationship with neighbors (10-point scale), online privacy infringement and personal information leakage instability (10-point scale), instability in the spread of high-risk new infectious disease (e.g., Middle East Respiratory Syndrome and Coronavirus) (10-point scale), economic recession and growth slowdown instability (10-point scale), environmental destruction and natural disaster instability (10-point scale), political and international relations (e.g., North Korea) instability (10-point scale), crime instability such as abuse and violence (10-point scale), social safety net vulnerability instability (10-point scale), low fertility and aging instability (10-point scale), instability in conflicts between classes, groups, and generations (10-point scale), your and your family’s experience of being a victim of a crime over the past year (yes or no), awareness of mental health promotion services provided by public health centers and/or mental health promotion centers (yes or no), and experiences of using mental health promotion services provided by public health centers and/or mental health promotion centers (yes or no).

The alcohol use disorders identification test (AUDIT) [31] is an alcohol use disorder screening test developed by the World Health Organization for the purpose of pre-screening drinkers at risk and reducing harmful effects through intervention in diseases that may be caused by excessive drinking as soon as possible. The AUDIT consisted of 10 items (total score is 40 points): 0 to 15 points were classified as normal drinkers, 16 to 19 points were high-risk drinkers, and 20 points or more were classified as alcohol use disorder. Self-esteem was measured using the Self Esteem Scale (SES) [32] developed by Rodenburg (1965). The SES consisted of 10 items (total score is 40 points), and a lower score was interpreted as lower self-esteem.

### 2.3. Development of Machine Learning Using Stacking Ensemble

This study used SVM and ensemble learning (i.e., random forest, LightGBM, and Adaboost) as the base model (single model). The first goal of this approach was to compare the predictive performance (accuracy) of the single model (base model), because previous studies [13,17,18,21,22,33], which tried to predict diseases using single machine learning, commonly used them and reported them as highly-accurate models. The second goal was to explore the stacking model with the best predictive performance by combining different base models and the meta model.

#### 2.3.1. Base Model: Support Vector Machine (SVM)

**Figure 1:**
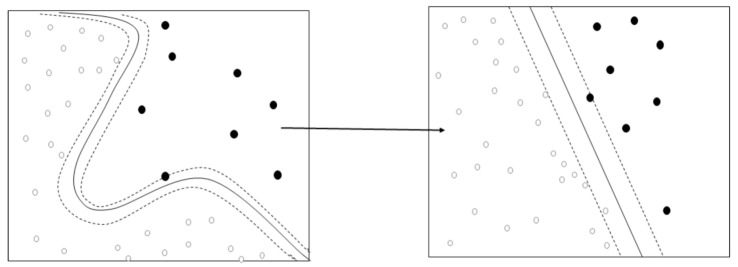
Concept of hyperplane in SVM [35].

SVM is a machine learning algorithm that finds the optimal decision boundary through linear separation that optimally separates the hyperplane [33]. SVM solves the nonlinear problem related to the input space (e.g., 2D) by transforming it into a high-dimensional feature space. For example, A = [a, d] and B = [b, c] are not linearly separable in 2D, however, when they are mapped in 3D, they can have a linearly separable feature. Thus, when adequate nonlinear mapping is conducted to a sufficiently large dimension, data with two classes can always be separated in the maximum-margin hyperplane (Figure 1). This separation boundary maximizes the separation between the two classes, and the training data closest to this boundary is defined as a support vector. Since SVM can model complex nonlinear decision-making domains, it is more accurate than other machine learning techniques and is less likely to cause an overfitting issue, which are advantages of this method [34,35]. This study chose the Gauss function (radial basis function), using parameter C (unit cost), for the SVM’s algorithm.

#### 2.3.2. Base Model: Random Forest

**Figure 2:**
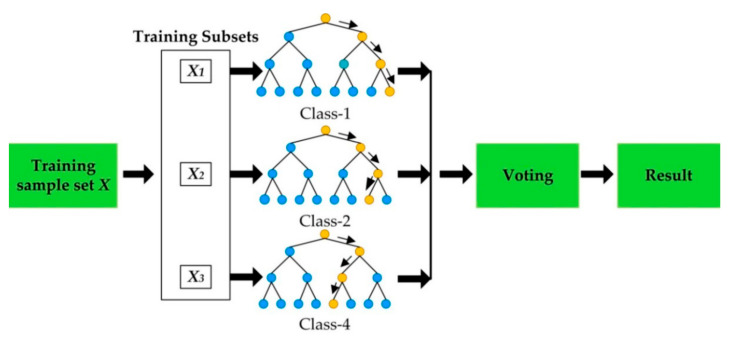
The concept of random forest [37].

Random forest is an algorithm that randomly learns multiple decision trees. It repeats random sampling for predictors and observations to create multiple decision trees. After obtaining prediction categories from numerous decision trees, the final category prediction is determined by a majority vote method. It can iteratively build independent decision trees by giving randomness to decision tree formation. This method can reduce prediction errors and it uses bootstrapping for random selection of predictors and observations [36]. In this study, 30 was the number of maximum leaf nodes, 10 was the maximum depth of tree, and 500 was the number of decision trees for fitting that were used as hyperparameters of random forest. The concept of random forest is presented in Figure 2.

#### 2.3.3. Base Model: LightGBM

LightGBM algorithm is a high-performance algorithm based on a decision tree algorithm and is mainly used for machine learning in order to rank or classify. GBM is inefficient in terms of training speed and memory consumption when it is applied to big data containing high-dimensional variables, which is a shortfall. To overcome this disadvantage, Microsoft introduced LightGBM, which rapidly calculates information gain using a portion of the data using Gradient-based One-Side Sampling (GOSS) and reduces features using exclusive feature bundling (EFB), in 2017 [38]. LightGBM splits the tree leaf-wise, unlike other boosting algorithms that split based on the depth or level of trees based on a decision tree algorithm. Therefore, when growing on the same leaf in LightGBM, the leaf-wise algorithm can reduce the loss better than the level-wise algorithm. In this study, learning rate for each lightGBM = 0.3, regularization term on weights = 0.1, colsample, subsample ratio of columns = 0.8, subsample ratio of the training instances = 0.8 were used as hyperparameters of lightGBM.

#### 2.3.4. Base Model: Adaboost

Adaboost is a learning technique that ultimately generates a strong classifier by iteratively training very weak classifiers using samples from two classes. It trains weak classifiers by giving the same weight to all samples and improves the performance of weak classifiers by increasing the weight of samples that were determined to be misclassified in the basic classifier as the steps progress. The concept of Adaboost’s algorithm is presented in Equation (1). In this study, learning rate for each Adaboost = 0.3, regularization term on weights = 0.1, colsample, subsample ratio of columns = 0.8, subsample ratio of the training instances = 0.8 were used as hyperparameters of Adaboost.

Given: $\left(x_{1},y_{1}\right),\;\ldots \;,\left(x_{m},y_{m}\right)$ where $x_{i}\in X,y_{i}\in \left\{-1,+1\right\}.$

Initialize: $D_{1}\left(i\right)=1/m\mathrm{for}\;i=1,\ldots \;,m.$

For t=1,… ,T:

- Train weak learner using distribution $D_{t}$.
- Get weak hypothesis $h_{t}\;:\;X\to \left\{-1,+1\right\}.$
- Aim: select $h_{t}$ with low weighted error:
  $\varepsilon_{t}=Pr_{i\sim D_{t}}\left[h_{t}\left(x_{i}\right)\neq y_{i}\right].$
- Choose $\alpha_{t}=\frac{1}{2}\ln\left(\frac{1-\varepsilon_{t}}{\varepsilon_{t}}\right).$
- Update, for i=1,… ,m:
  $$D_{t+1}\left(i\right)=\frac{D_{t}\left(i\right)\exp\left(-\alpha_{t}y_{i}h_{t}\left(x_{i}\right)\right)}{Z_{t}}$$
  where $Z_{t}$ is a normalization factor (chosen so that $D_{t+1}$ will be a distribution).

Output the final hypothesis:

(1) $$H\left(x\right)=\mathrm{sign}\left(\overset{T}{\underset{t=1}{\sum }}\alpha_{t}h_{t}\left(x\right)\right).$$

### 2.4. Meta Model: XGBoost

This study predicted anxiety disorder in old age through the stacking ensemble technique. The stacking ensemble is better than recent single predictive models in terms of generalization and robustness, and it has been used for classification and prediction in various fields [16,17,18]. This method generates a new model by combining different various models as if stacking them in multiple layers, and it goes through two stages (base and meta). It improves the performance of the final model by taking the strength of each model and compensating for the weakness of each model [39].

**Figure 3:**
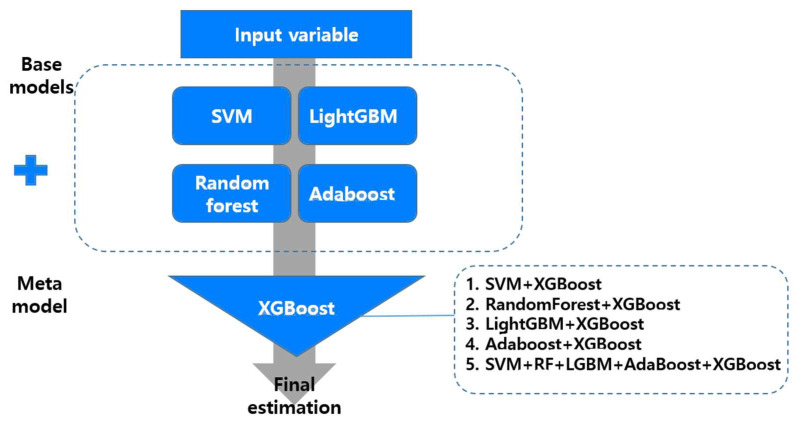
Process flow diagram for predictive models.

This study used SVM, random forest, LightGBM, and Adaboost for the base model, a single predictive model, while using XGBoost algorithm for the meta model. XGBoost is a method to increase the reliability of the base model while maximizing its stability [40]. Lin et al. (2018) [41] also reported that the accuracy was improved compared to a single predictive model when applying XGBoost to a stacking ensemble model. Thus, this study also used XGBoost as a meta model. In this study, learning rate for each tree = 0.3, regularization term on weights = 0.001, colsample, subsample ratio of columns = 0.8, subsample ratio of the training instances = 0.8, maximum depth of tree = 10 were used as hyperparameters of XGBoost. Finally, this study developed four base models and five stacking ensemble models (SVM + XGBoost, random forest + XGBoost, LightGBM + XGBoost, Adaboost + XGBoost, and SVM + RF + LGBM + AdaBoost + XGBoost) to predict anxiety disorders in old age (Figure 3).

### 2.5. Validation of Model’s Predictive Performance

The predictive performance of the nine developed machine learning models was validated using seven-fold cross-validation. This method randomly divides the entire sample into seven groups of equal size, and treats one group as a validation dataset and others as training datasets. It repeats this process seven times to prove the validity of the learning. This study used accuracy, precision, recall, and F1-score as indices to evaluate predictive performance. The calculation formula of each evaluation index is presented in Equation (2).

True positive = It is actually an anxiety disorder, and the predicted outcome is an anxiety disorder.(2)False negative = It is actually an anxiety disorder, but the predicted outcome is normal.False positive = It is actually normal, and the predicted outcome is an anxiety disorder.True negative = It is actually normal, and the predicted outcome is normal.Recall = TP/(TP + FN)Precision = TP/(TP + FP)Accuracy = (TP + TN)/(TP + FP + FN + TN)F1-score = 2 * (Recall * Precision)/(Recall + Precision)

This study assumed that a model with the highest F1-score was the best predictive performance. If the F1-score was the same, a model with the highest recall was assumed as the best model. All analyses were performed using Python version 3.8.6 (https://www.python.org (accessed on 21 June 2021).

## 3. Results

### 3.1. General Characteristics of Subjects

The general characteristics of the subjects are presented in Table 1. The prevalence of the anxiety disorder among the elderly in South Korea was 17.2%. The results of the chi-square test showed that the elderly with a depressive disorder and the elderly without a depressive disorder had significant (*p* < 0.05) self-esteem, subjective loneliness, subjective satisfaction with help from neighbors, subjective satisfaction of the medical service of the region, regular club activities, the experience of suicidal urge over the past year, subjective trust satisfaction with neighbors, subjective frequency of communication with neighbors and friends, alcohol use disorder, subjective satisfaction in the living environment of the neighborhood, subjective satisfaction in the safety level of the neighborhood, age, subjective frequency of communication with other family members, the highest level of education, mean monthly household income, subjective level of discussion and consultation with neighbors and friends, your and your family’s experience of being a victim of a crime over the past year, income instability, instability of preparation for old age, living safety instability, physical health instability, mental health instability, family relationship and dissolution instability, instability in child education, family support, and caregiving, instability in relationship with neighbors, instability in the spread of high-risk new infectious disease, economic recession and growth slowdown instability, crime instability such as violence, social safety net vulnerability instability, low fertility and aging instability, and instability in conflicts between classes, groups, and generations.

### 3.2. Comparing the Accuracy of Predictive Models for the Anxiety Disorder in Old Age

**Figure 4:**
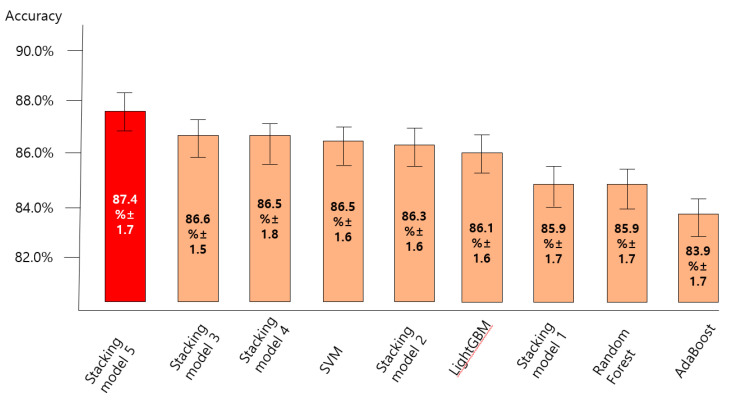
Comparing the accuracy of nine machine learning models for predicting anxiety disorders in old age.

**Figure 5:**
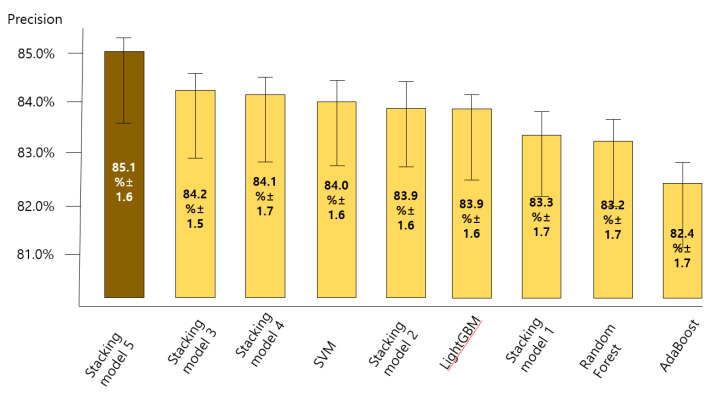
Comparing the precision of nine machine learning models for predicting anxiety disorders in old age.

**Figure 6:**
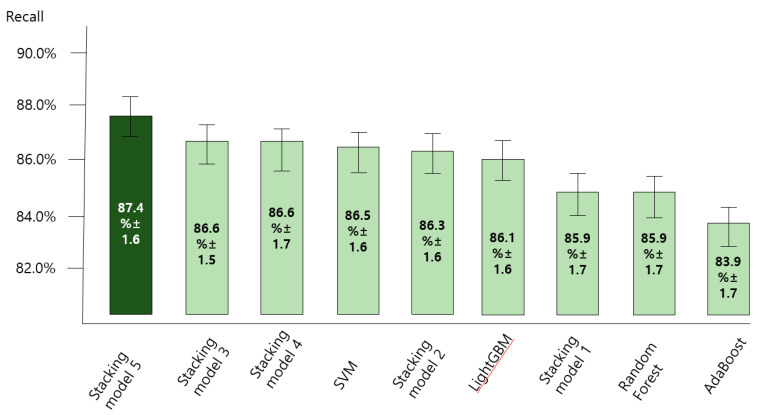
Comparing the recall of nine machine learning models for predicting anxiety disorders in old age.

**Figure 7:**
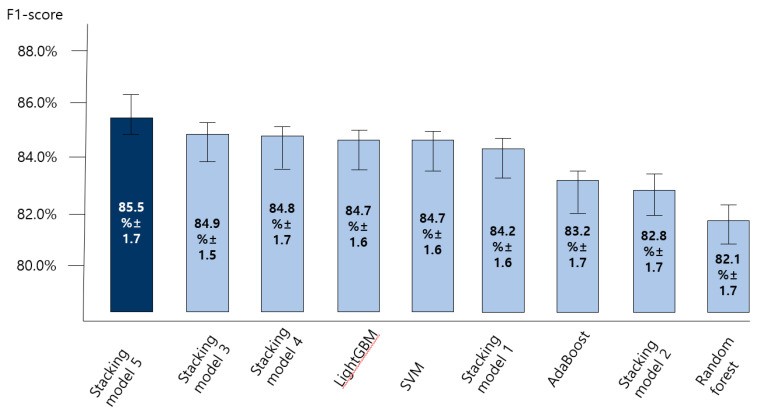
Comparing the F1 score of nine machine learning models for predicting anxiety disorders in old age.

Figure 4, Figure 5, Figure 6 and Figure 7 show the predictive performance (accuracy, precision, recall, and F1-score) of nine machine learning models for prediction of the anxiety disorder in old age, respectively. The analysis results confirmed that the predictive performance of the “SVM + RF + LGBM + AdaBoost + XGBoost model (stacking ensemble: accuracy 87.4%, precision 85.1%, recall 87.4%, and F1-score 85.5%)” was the best.

Stacking model 1 = SVM + XGBoost; Stacking model 2 = RandomForest + XGBoost; Stacking model 3 = LightGBM + XGBoost; Stacking model 4 = Adaboost + XGBoost; Stacking model 5 = SVM + RF + LGBM + AdaBoost + XGBoost.

Stacking model 1 = SVM + XGBoost; Stacking model 2 = RandomForest + XGBoost; Stacking model 3 = LightGBM + XGBoost; Stacking model 4 = Adaboost + XGBoost; Stacking model 5 = SVM + RF + LGBM + AdaBoost + XGBoost.

Stacking model 1 = SVM + XGBoost; Stacking model 2 = RandomForest + XGBoost; Stacking model 3 = LightGBM + XGBoost; Stacking model 4 = Adaboost + XGBoost; Stacking model 5 = SVM + RF + LGBM + AdaBoost + XGBoost.

Stacking model 1 = SVM + XGBoost; Stacking model 2 = RandomForest + XGBoost; Stacking model 3 = LightGBM + XGBoost; Stacking model 4 = Adaboost + XGBoost; Stacking model 5 = SVM + RF + LGBM + AdaBoost + XGBoost.

### 3.3. Exploring Predictors and High-Risk Groups for the Anxiety Disorder in Old Age

**Figure 8:**
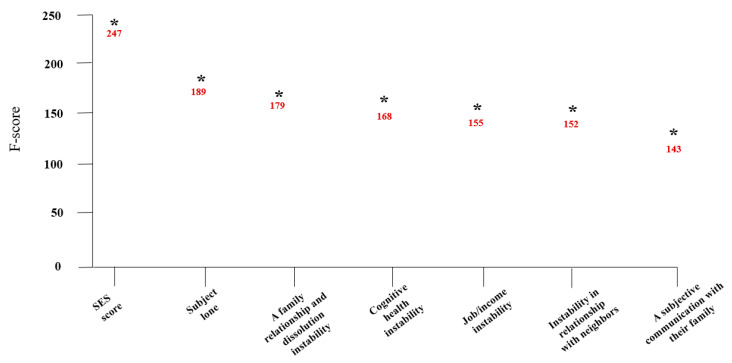
The importance of variables in the prediction model for anxiety disorder in old age (only the top seven variables are presented).

The feature importance of the SVM + RF + LGBM + AdaBoost + XGBoost model, the final model for predicting the anxiety disorder in the elderly in South Korea, is presented in Figure 8. In this model, subjective loneliness, SES, subjective family relations, instability in family support and caregiving, subjective frequency of communication with family, family relationship and dissolution instability, and your and your family’s experience of being a victim of a crime over the past year were identified as the seven major variables with high weight. Among them, subjective loneliness was the most important factor in the final model.

**Figure 9:**
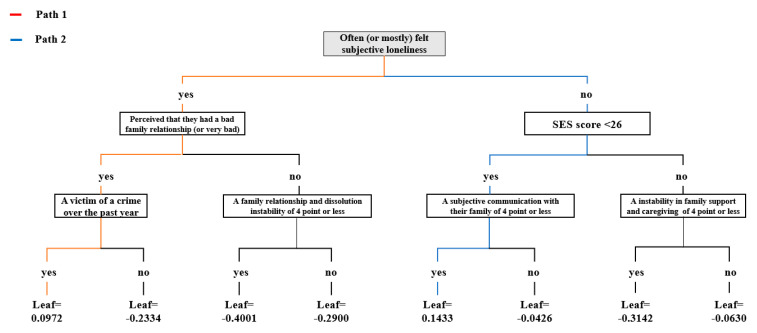
A tree plot that presents seven variables with high weight in the importance using the decision tree visualization.

Figure 9 shows a tree plot that presents seven variables with high weight in importance, using the decision tree visualization. The value of the leaf node represents the logistic function probability score (LFP-score). A positive number refers to the probability of having a depressive disorder, and a negative number indicates the probability of not having an anxiety disorder. There were two paths for predicting anxiety disorder in old age (Table 2). The first path with the highest predictive probability of an anxiety disorder was the elderly who often (or mostly) felt subjective loneliness, had an SES score of 26 or less, and had a subjective communication with their family of 4 or less (on a 10-point scale). The second path was the elderly who sometimes felt subjective loneliness, perceived that they had a bad family relationship (or very bad), and were victims of a crime, or had a family member who was a victim of a crime over the past year.

## 4. Discussion

This study compared the predictive performance (accuracy) of nine machine learning algorithms to predict anxiety disorders in the elderly in South Korea and confirmed that the SVM + RF + LGBM + AdaBoost + XGBoost model had the best predictive performance. SVM + RF + LGBM + AdaBoost + XGBoost, LightGBM + XGBoost, and Adaboost + XGBoost, among the stacking ensemble models in this study, had higher accuracy, precision, recall, and F1-score than single predictive models. The results agreed with previous studies [42,43], which reported that the root-mean-square error (RMSE) of the stacking ensemble model was lower than that of the single machine learning model. In particular, Byeon (2021) [43] showed that the stacking ensemble model had a higher index of agreement (IA) and variance of errors (Ev), in addition to accuracy, than the single machine learning model, which implied that the predictive performance of the stacking ensemble model could be higher than that of the single predictive model for structured data such as examination data. However, in this study, the F1-score of SVM was 0.5% higher than that of SVM + XGBoost, which suggested that the stacking ensemble model could perform worse than a single machine learning model depending on the combination of a base model and a meta model. Therefore, futures studies shall explore the stacking ensemble model with the best performance in community examination data by combining various base models and meta models including unsupervised learning (e.g., clustering), in addition to boosting to prove the performance of the stacking ensemble. 

Another finding of this study was that subjective loneliness, SES, subjective family relations, instability in family support and caregiving, subjective frequency of communication with family, family relationship and dissolution instability, and your and your family’s experience of being a victim of a crime over the past year were independent risk factors for predicting an anxiety disorder in the elderly living in South Korea. Green et al. (2013) [44] conducted a cohort study on Scottish people and reported that socioeconomic differences such as low educational level and low level of income were significantly related to anxiety in old age. However, socioeconomic factors were not significantly related to anxiety in this study, while the effects of family factors such as anxiety about the dissolution of family relations and anxiety due to family support or caregiving were significantly higher. It is believed that the difference from previous studies is due to the characteristics of the elderly of South Korea who value traditional family relationships. The structure and value of family relations have been changed in the past 30 years in South Korea as the traditional family system has been breaking up the nuclear family through the process of rapid industrialization and urbanization [45]. As the range of the elderly’s social life has been reduced, their interests and contacts have shifted from society to their families [45]. As a result, family relationships have a significant impact on the psychological health of the elderly [45]. Although it is impossible to conclude that the frequency of communication with family members living together can sufficiently determine the emotional support for the elderly, the results of this study implied that the emotional support obtained from other family members or people around them can alleviate anxiety in old age. Since not enough studies have evaluated factors influencing the anxiety of the elderly, more epidemiological studies are required to understand the characteristics of anxiety in old age.

Previous studies [26,27,28,29,30] that identified factors related to the anxiety of the elderly only tried to identify individual factors of depression using regression analysis. Therefore, they are limited in identifying multiple risk factors for anxiety. This community-based epidemiologic study identified multiple risk factors using the decision tree visualization of the stacking ensemble model. The results of this study showed that the elderly who often (or mostly) felt subjective loneliness, had an SES score of 26 or less, and had a subjective communication with their family of 4 or less (on a 10-point scale) were the group with the highest risk of anxiety disorder. 

When people get older, they experience social isolation and loneliness due to separation from their children, retirement from work, and the death of people around them (e.g., spouse, family, and friends). If this loneliness persists, they will be more likely to develop depressive and anxiety disorders [46]. Statistics Korea (2021) [47] forecasted that South Korea will enter a super-aged society in 2026, which means that one in four people will be elderly. It is five years before 2026, and one in five elderly people (17.2%) are suffering from an anxiety disorder. However, there are not sufficient policy measures for identifying anxiety disorders in the elderly soon and managing high-risk groups compared to cognitive disorders such as chronic diseases or dementia. Since early detection and preventive treatment are important for mental disorders such as anxiety, it will be necessary to continuously monitor the elderly who perceive that they have a bad relationship with their family, subjectively experience a lot of loneliness, and frequently feel anxious about family relationships and dissolution to prevent anxiety disorders in old age based on the results of this study from the community level (or primary medical care level). Furthermore, since almost no studies have identified multiple risk factors for anxiety in old age, more epidemiological studies are required to continuously identify multiple risk factors for anxiety in old age.

The importance of this study was that this study analyzed complex factors of anxiety such as individual characteristics, family factors, and social environment using epidemiological data that can represent the elderly living in a local community. When developing a predictive model using medical data, the critical elements are to explain (interpret) the results and to secure high accuracy. This study presented the derived key predictors using decision tree visualization, which added the possibility of explanation. The decision tree visualization technique of the ensemble machine, presented in this study, is meaningful because it presents an application case of interpretable AI using structured data and the grounds of its use. Future studies are needed to develop X-AI or transparent AI using various methods based on structured data in order to explain the judgment of AI in a form that medical personnel can understand based on the interpretable AI case of this study.

The limitations of this study are as follows. First, this study could not identify the detailed types of anxiety disorders due to the nature of the epidemiological investigation using the anxiety disorder screening test. Future studies are needed to classify the types of anxiety disorders into a generalized anxiety disorder, phobia disorder, panic disorder, and obsessive-compulsive disorder using medical diagnosis and to explore risk factors according to the type. Second, although social networks such as the number of close friends to meet are important for anxiety disorder in old age, this epidemiological study did not investigate social networks. Third, this study used a secondary source, the KPA Survey conducted in 2015. Therefore, there is a possibility that there is a difference between the general characteristics of older adults surveyed in 2015 and those of older adults in 2021. Consequently, the results of this study should be interpreted carefully. Fourth, since this study is a cross-sectional study, even if risk factors for anxiety disorder are identified, their causal relationships cannot be argued. Additional longitudinal studies are required to prove the causal relationship between the multiple risk factors for anxiety disorder in old age identified in this study.

## 5. Conclusions

The results of this study indicated that it will be necessary to continuously monitor subjective loneliness, SES, subjective family relations, instability in family support and caregiving, subjective frequency of communication with family, family relationship and dissolution instability, and your and your family’s experience of being a victim of a crime over the past year to prevent and screen anxiety disorders in the elderly living in a local community as soon as possible. Furthermore, it is necessary to establish a community-based mental health policy that can identify elderly groups with high anxiety risks based on multiple risk factors and manage them constantly.

## Figures and Tables

**Table 1:** The general characteristics of the subjects: univariate analysis.

Variables	Anxiety Disorder		*p*
	No (n = 1290)	Yes (n = 268)	
Residential area			0.621
Urban	981 (83.1)	200 (16.9)	
Rural	309 (82.0)	68 (18.0)	
Highest level of education			<0.001
Middle school graduation or less	804 (79.6)	206 (20.4)	
High school graduation or more	486 (88.7)	62 (11.3)	
Mean monthly household income			0.001
<KRW 2 million	739 (80.1)	184 (19.9)	
≥KRW 2 million and <KRW 3 million	248 (84.9)	44 (15.1)	
≥KRW 3 million	303 (88.3)	40 (11.7)	
Subjective loneliness			<0.001
Very rare	653 (95.1)	34 (4.9)	
Occasionally lonely	530 (82.9)	109 (17.1)	
Often lonely	100 (47.6)	110 (52.4)	
Mostly lonely	7 (31.8)	15 (68.2)	
Subjective satisfaction with help from neighbors			<0.001
Yes	915 (85.7)	153 (14.3)	
No	375 (76.5)	115 (23.5)	
Self-esteem, the experience of suicidal urge over the past year			<0.001
Yes	66 (47.1)	74 (52.9)	
No	1224 (86.3)	194 (13.7)	
Subjective trust satisfaction with neighbors			<0.001
Yes	1153 (85.7)	193 (14.3)	
No	137 (64.6)	75 (35.4)	
Alcohol use disorder			<0.001
Normal drinker	685 (83.8)	132 (16.2)	
High-risk drinker	225 (80.9)	53 (19.1)	
Alcohol use disorder	5 (38.5)	8 (61.5)	
Subjective satisfaction of the living environment of the neighborhood			<0.001
Yes	1032 (85.9)	169 (14.1)	
No	258 (72.3)	99 (27.7)	
Subjective satisfaction of the safety level of the neighborhood			<0.001
Yes	1104 (85.9)	181 (14.1)	
No	186 (68.1)	87 (31.9)	
Subjective satisfaction of the medical service of the region			0.007
Yes	947 (84.4)	175 (15.6)	
No	343 (78.7)	93 (21.3)	
Regular club activities			0.001
Yes	421 (87.7)	59 (12.3)	
No	869 (80.6)	209 (19.4)	
Your and your family’s experience of being a victim of a crime over the past year			<0.001
Yes	28 (56.0)	22 (44.0)	
No	1206 (84.5)	221 (15.5)	
Awareness of mental health promotion services provided by public health centers and/or mental health promotion centers			0.662
Yes	330 (82.1)	72 (17.9)	
No	960 (83.0)	196 (17.0)	
Experiences of using mental health promotion services provided by public health centers and/or mental health promotion centers			0.372
Yes	95 (79.8)	24 (20.2)	
No	1195 (83.0)	244 (17.0)	
Age, mean ± SD	67.77 ± 5.53	69.01 ± 5.50	0.001
Subjective frequency of communication with other family members, mean ± SD	6.40 ± 1.53	5.22 ± 1.91	<0.001
Subjective frequency of communication with neighbors and friends, mean ± SD	6.18 ± 1.56	5.23 ± 1.79	<0.001
Self esteem scale, mean ± SD	29.28 ± 3.21	26.13 ± 3.35	<0.001
Subjective frequency of communication with neighbors and friends (10 points scale)	5.90 ± 1.54	5.16 ± 1.84	<0.001
Job/income instability (10-point scale)	5.07 ± 1.84	5.86 ± 2.14	<0.001
Instability of preparation for old age (10-point scale)	6.29 ± 2.16	7.27 ± 1.71	<0.001
Living safety instability (10-point scale)	4.66 ± 2.04	5.34 ± 1.87	<0.001
Physical health instability (10-point scale)	6.17 ± 2.14	7.02 ± 2.08	<0.001
Cognitive health instability (10-point scale)	4.86 ± 2.11	6.33 ± 1.95	<0.001
Family relationship and dissolution instability (10-point scale	3.79 ± 2.23	5.31 ± 2.14	<0.001
Instability in family support and caregiving (10-point scale)	3.71 ± 2.28	4.45 ± 2.47	<0.001
Instability in relationship with neighbors (10-point scale)	3.45 ± 2.17	4.27 ± 2.15	<0.001
Online privacy infringement and personal information leakage instability (10-point scale)	3.76 ± 2.37	3.49 ± 2.31	0.094
Instability in the spread of high-risk new infectious disease (10-point scale)	6.35 ± 1.96	6.74 ± 1.66	0.003
Economic recession and growth slowdown instability (10-point scale)	6.42 ± 1.87	6.69 ± 1.80	0.032
Environmental destruction and natural disaster instability (10-point scale)	5.47 ± 1.88	5.66 ± 1.61	0.130
Political and international relations instability	5.63 ± 1.92	5.28 ± 2.02	0.993
Crime instability such as abuse and violence (10-point scale)	5.28 ± 2.02	5.73 ± 2.03	0.001
Social safety net vulnerability instability (10-point scale)	5.37 ± 2.03	5.79 ± 1.97	0.002
Low fertility and aging instability (10-point scale)	5.38 ± 2.12	5.75 ± 2.06	0.010
Instability in conflicts between classes, groups, and generations (10-point scale)	5.03 ± 2.02	5.33 ± 2.01	0.027

**Table 2:** A path for predicting anxiety disorder in old age.

Path	Characteristics	LFP-Score
1	The elderly who often (or mostly) felt subjective loneliness, had an SES score of 26 or less, and had a subjective communication with their family of 4 or less (on a 10-point scale)	0.14
2	The elderly who sometimes felt subjective loneliness, perceived that they had a bad family relationship (or very bad), and were victims of a crime, or had their family member who was a victim of a crime over the past year	0.09

LEP-score = logistic function probability score.

## Data Availability

Restrictions apply to the availability of these data. Data was obtained from Korea Institute for Health and Social Affairs and are available [from the Korea Institute for Health and Social Affairs/https://www.kihasa.re.kr/en (accessed on 21 June 2021)] with the permission of Korea Institute for Health and Social Affairs.
